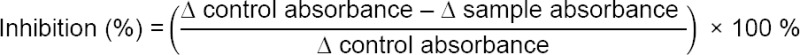# Study of HMG-CoA Reductase Inhibition Activity of the Hydrolyzed Product of Snakehead Fish (*Channa striata*) Skin Collagen with 50 kDa Collagenase from *Bacillus licheniformis* F11.4

**DOI:** 10.3797/scipharm.ISP.2015.01

**Published:** 2016-02-14

**Authors:** Agnes Virginia, Heni Rachmawati, Catur Riani, Debbie S. Retnoningrum

**Affiliations:** School of Pharmacy, Institut Teknologi Bandung, Ganeca 10, 40132 Bandung, Indonesia

**Keywords:** *Bacillus licheniformis* F11.4, HMGR inhibitor, Snakehead fish skin collagen, Collagenase

## Abstract

Bioactive peptides produced from enzymatic hydrolysis fibrous protein have been proven to have several biological activities. Previous study showed that the hydrolysis product of snakehead fish skin collagen with 26 kDa collagenase from *Bacillus licheniformis* F11.4 showed HMG-CoA (HMGR) inhibition activity. The aim of this research was to determine the ability of the hydrolysis product produced from snakehead fish skin collagen hydrolysed by 50 kDa collagenase from *B. licheniformis* F11.4 in inhibiting HMGR activity. Snakehead fish skin collagen was extracted using an acid method and collagenase was produced from *B. licheniformis* F11.4 using half-strength Luria Bertani (LB) medium containing 5% collagen. Crude collagenase was concentrated and fractionated using the DEAE Sephadex A-25 column eluted with increasing gradient concentrations of NaCl. Collagen, collagenase, and fractions were analyzed using SDS-PAGE and collagenolytic activity was analyzed by the zymography method. Collagenase with 50 kDa molecular weight presented in fraction one was used to hydrolyze the collagen. The reaction was done in 18 hours at 50°C. The hydrolysis product using 3.51 μg collagen and 9 ng collagenase showed 25.8% inhibition activity against pravastatin. This work shows for the first time that the hydrolysis product of snakehead fish skin collagen and 50 kDa collagenase from *B. licheniformis* F11.4 has potential as an anticholesterol agent.

## Introduction

Marine organisms are great sources of various biofunctional components and starting materials for protein derived bioactive peptides [1]. Bioactive peptides are specific protein fragments consist of 3–20 amino acids that have diverse physiological functions [2]. Their activities depend on their amino acid sequences and compositions. The activities include opioid, antibiotic, antithrombotic, immunomodulation and antihypertensive activity [3].

One of the methods to obtain bioactive peptides is enzymatic hydrolysis [4]. Enzymatic hydrolysis consists of two main components that are protein and protease. Collagen is the sources of protein that can be found in fish skin and it is the major structure protein of connective tissues. Fibrillar collagens, the most abundant, include type I, II, III, V and XI [5]. Type I collagen is the most abundant collagen and is present in tendon, bone, ligament, while type II collagen is the main component of cartilage and type III collagen is present in skin and blood vessels. Type I collagen is a heterotrimer composed of two α1(I) chains and one α2(I) chain which are connected with hydrogen bond, forming triple helix structure. Type II collagen is a homotrimer composed of three α1(II) chains. Type III collagen is a homotrimer composed of three α1(III) chains. In all three types of collagen, each of the chain is about 1000 amino acids in length [5]. Collagen polypeptide chains comprise of three main amino acids, glycine, proline and hydroxyproline [6]. Collagen used in this experiment was type I collagen which was originated from fish skin.

Collagenase is an enzyme that has capability to hydrolyze triple helix structure of collagen. Collagenase can be obtained from vertebrate organs extracts and bacteria. Collagenase from bacteria can degrade not only native collagen but also degraded collagen, while collagenase from vertebrates can only degrade native collagen [7]. Enzyme cleaves the Gly^775^-Ile^776^ bond of the α1 chain and the Gly^775^-Leu^776^ bond of the α2 chain [6]. One of bacteria that produce collagenase is *Bacillus licheniformis* F11.4 [8].

Bioactive peptides resulted from hydrolysis product of collagen and collagenase was tested for its anticholesterol activity as HMGR inhibitor. HMGR is an enzyme that converts HMG-CoA into cholesterol in cholesterol biosynthesis. As HMGR enzyme is inhibited, the synthesis of cholesterol will also be inhibited. Cholesterol is a major biological component of biological membranes and a precursor for hormones [9]. High level of total cholesterol in blood can cause atherosclerosis that can lead to heart problems, including stroke. Therefore, anticholesterol drugs have been intensively studied.

## Results and Discussion

Enzymatic hydrolysis consists of two main components that are protein and protease. In this research, the protein used was snakehead fish skin collagen and the protease used was collagenase from *B. licheniformis* F11.4. Collagen was extracted from snakehead fish skin using acid method. The weight of the snakehead fish skin used for extraction was 58 gram.

**Fig. 1:**
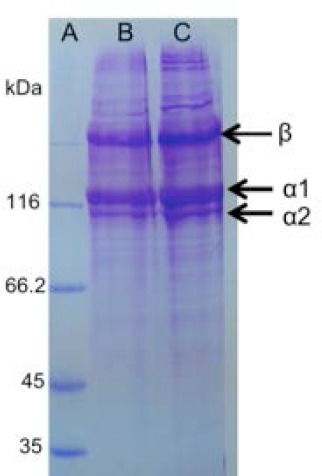
SDS-PAGE analysis of snakehead fish skin collagen. A: markers, B: non-reduction, C: reduction. Arrows showed collagen consisted of α1, α2 and β bands.

SDS-PAGE analysis showed that collagen had α1 and α2 chains with respective molecular weight of 117.07 kDa and 107.31 kDa as the major component, and β chain as a dimerization product of α chain (Fig.1). The concentration of extracted collagen using Bradford’s method was 0.234 mg/mL.

**Fig. 2:**
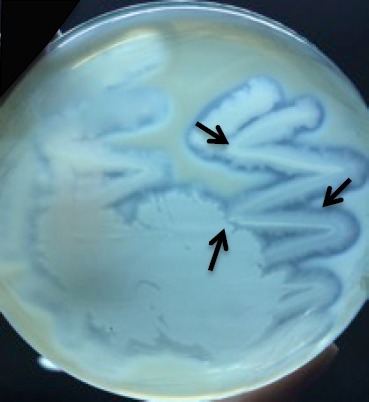
The proteolysis activity of B. licheniformis F11.4. Arrows showed clear areas surrounding the bacterium.

After collagen was obtained, the next step was production and isolation of collagenase from *B. licheniformis* F11.4. Bacterial cells that were used for producing collagenase was first confirmed its proteolysis activity. The result showed formation of clear areas that surrounded the bacterial colony in Muller Hinton Broth (MHB) media containing 2% collagen (Fig.2). This finding proved that the bacterium produced extracellular protease.

**Fig. 3:**
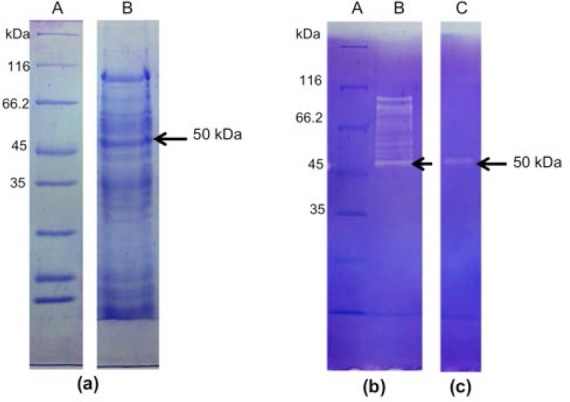
SDS-PAGE analysis and zymogram of collagenase. (a) Electrophoretogram of crude collagenase, (b) zymogram of crude collagenase, (c) zymogram of fraction one. A: markers, B: crude collagenase, C: fraction one. Arrows showed 50 kDa collagenase used for hydrolysis reactions.

The bacterium culture that has been confirmed for its activity was used to produce collagenase. The condition of collagenase production was controlled by preparing a starter of the bacterium in LB media until it reached OD_600_ of 0.7-0.8. The starter was sub-cultured into half-strength of LB media containing 5% collagen to achieve OD_600_ of 0.8-0.9. Crude collagenase was concentrated and analyzed using SDS-PAGE and zymography method to confirm its collagenolytic activity (Fig.3a and Fig.3b). SDS-PAGE analysis confirmed that the bacterium produced collagenase target that had molecular weight of 50 kDa. Zymogram presented there was active collagenase band at 50 kDa and this collagenase was used to hydrolyze the collagen. This 50 kDa collagenase was fractionated using DEAE Sephadex A-25 column. Each fraction was analyzed using zymography method to determine which fractions contained single band of 50 kDa collagenase. Fraction one consisted 50 kDa collagenase (Fig.3c) and its concentration was 0.9 μg/mL

**Fig. 4:**
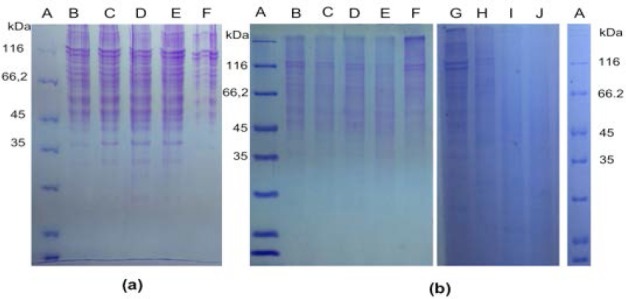
SDS-PAGE analysis of hydrolysis products. (a) first optimization, (b) second optimization. The amount presented in collagen (μg) / collagenase (ng). A: markers, B: 5.85/4.5, C: 5.85/9, D: 5.85/13.5, E: 5.85/0, F: 1.17/0. (b) A: markers, B: 3.51/27, C: 3.51/22.5, D: 3.51/18, E: 3.51/13.5, F: 3.51/0, G: 0.702/0, H: 3.51/0, I: 3.51/4.5, J: 3.51/9.

Optimization was performed to determine the optimum amount of collagen and collagenase used in hydrolysis reaction. First optimization was done using 5.85 μg of collagen with addition of 13.5, 9 and 4.5 ng of collagenase. The formation of new bands or decrease in the intensity of initial bands in SDS-PAGE analysis indicated hydrolysis reaction. In the first optimization, there was no hydrolysis reaction (Fig.4a). Therefore, the second optimization was done by decreasing the amount of collagen to 3.51 μg with addition of 27, 22.5, 18, 13.5, 9 and 4.5 ng of collagenase (Fig.4b). The electrophoretogram showed the optimum amount for hydrolysis reaction was 3.51 μg of collagen with 9 ng of collagenase.

**Fig. 5:**
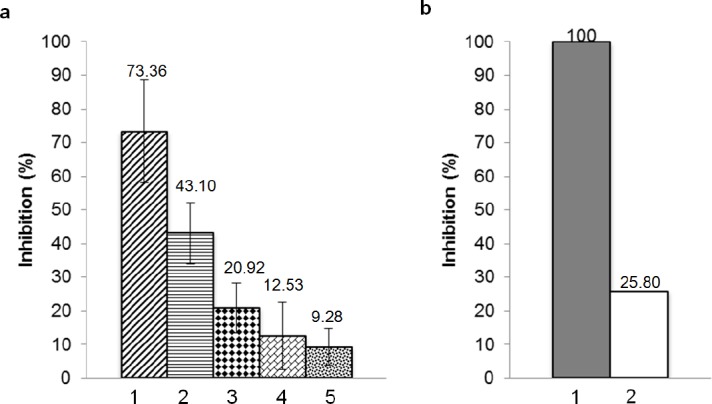
Inhibition percentage of hydrolysis product. (a) 1: pravastatin, 2: hydrolysis product, 3: collagen, 4: collagenase, 5: water. (b) Relative amount to pravastatin. 1: pravastatin 100%, 2: corrected inhibition percentage of hydrolysis product

HMGR inhibition activity of hydrolysed product from 3.51 μg of collagen and 9 ng of collagenase was analyzed in triplicate using HMG-CoA Reductase Assay Kit. The principle of this assay was measurement of NADPH absorbance at 340 nm at 37°C. Inhibition percentage was calculated at 600 seconds and it was presented in Fig.5a. The inhibition percentage oh hydrolysis product was measured by eliminating inhibition percentage of the components involved in the reaction, which were collagen, collagenase, and water. By assuming that pravastatin would give 100 percent HMGR inhibition activity, the inhibition activity of hydrolysis product was 25.8% (Fig.5b). In conclusion, the hydrolysis product of snakehead fish skin collagen with 50 kDa collagenase from *B. licheniformis* F11.4 had a potency to be developed as anticholesterol agent.

## Experimental

### Microorganism

The B. licheniformis F11.4 was a gift from Prof. Maggy T. Suhartono, Department of Food Technology, Institut Pertanian Bogor.

### Extraction of acid soluble snakehead fish skin collagen

Snakehead fish skin collagen was extracted using acid method with a slight modification [10]. All the preparative procedures were done at 25°C. The skin was soaked in 50% of ethanol (1:2) for 30 minutes, then washed with distilled water until pH 7. The skin was extracted with 0.1 N NaOH (1:10) for 6 hours to remove non-collagenous proteins, then washed with distilled water until pH 7. Insoluble matters were extracted with 0.5 M acetic acid (1:15) for 24 hours. The extract was filtered using 150 μm sieve. The residue was re-extracted with the same solution for 24 hours. Each viscous solution was mixed and salted out by adding mixture of 2.6 M NaCl and 0.05 M tris(hydroxymethyl) aminomethane. The resultant precipitate was obtained by centrifugation at 5,000 x g for 2 hours at 4°C and dissolved in 0.5 M acetic acid, dialyzed against 0.1 M acetic acid and distilled water for 24 hours.

### Confirmation of proteolysis activity, production, and fractionation of B. licheniformis F11.4 collagenase

Isolate of B. licheniformis F11.4 was cultured in LB media and incubated at 37°C, 150 rpm for 18 hours. Proteolysis activity of the bacterium was confirmed by inoculating it in the MHB media containing 2% of skim milk. The proteolysis activity was verified by clear areas surrounding the inoculum. The culture that has been confirmed was used to produce collagenase.

The bacterium was cultured in 4 mL LB media at 37°C at 150 rpm and then it was sub-cultured in 100 mL production media (1% NaCl, 0.5% triptone, 0.25% yeast extract, and 5% snakehead fish skin collagen) [9]. Supernatant was obtained by centrifugation at 4,500 x g for 20 minutes at 4°C and then it was concentrated using column. Crude concentrated collagenase was stored at 4°C.

Crude collagenase was fractionated using DEAE Sephadex A-25. The column was washed with 0.02 M phosphate buffer pH 7.0 at a flow rate of 0.5 mL/min. Protein fractions were eluted with a step wise of NaCl concentration 0; 0.125; 0.25; 0.5; 0.75 and 1 M in 0.02 M phosphate buffer pH 8.0 at a rate of 5 ml/min. The fractions with 50 kDa collagenase activity were pooled and concentrated. The pooled collagenase fractions were stored at 4°C.

### Molecular weight, protein determination, and zymography

Molecular weight was estimated by electrophoresis under denaturing polyacrylamide-SDS (SDS-PAGE) with 15% polyacrylamide gels. The standard molecular weight markers were beta-galactosidase (116 kDa), bovine serum albumin (66.2 kDa), ovalbumin (45 kDa), lactate dehydrogenase (35 kDa), REase Bsp98I (25 kDa), beta-lactoglobulin (18.4 kDa) and lysozyme (14.4 kDa).

Enzymatic activity in situ was determined by zymography method following SDS-PAGE in 15% acrylamide. For zymography analysis, collagen at 0.1% was incorporated into the gel. Gel was soaked in Triton X-100 2.5% for 1.5 hours and clear bands showed collagenolytic activity.

Protein concentration was measured by Bradford’s method (1976) using reagents consisted of 100 mg comassie brilliant blue (CBB) G-250 in 50 mL of ethanol 95% and 100 mL of phosphate acid 85% in 1 liter. Bovine serum albumin was used as the protein standard. Triplicate experiments were carried out for each measurement.

### Optimization the amount collagen and collagenase for hydrolysis reaction and HMGR inhibition activity assay

Optimization was done by varying the amount of collagen and 50 kDa collagenase. Hydrolysis reaction was done at 50°C for 18 hours and the reaction was stopped by incubating it at 90°C for 10 minutes. Hydrolyisis product was analyzed for its HMGR inhibition activity using HMG-CoA Reductase Assay Kit (Sigma). Inhibition percentage was measured using this formula: